# Dietary Fibre Consensus from the International Carbohydrate Quality Consortium (ICQC)

**DOI:** 10.3390/nu12092553

**Published:** 2020-08-24

**Authors:** Livia S. A. Augustin, Anne-Marie Aas, Arnie Astrup, Fiona S. Atkinson, Sara Baer-Sinnott, Alan W. Barclay, Jennie C. Brand-Miller, Furio Brighenti, Monica Bullo, Anette E. Buyken, Antonio Ceriello, Peter R. Ellis, Marie-Ann Ha, Jeyakumar C. Henry, Cyril W. C. Kendall, Carlo La Vecchia, Simin Liu, Geoffrey Livesey, Andrea Poli, Jordi Salas-Salvadó, Gabriele Riccardi, Ulf Riserus, Salwa W. Rizkalla, John L. Sievenpiper, Antonia Trichopoulou, Kathy Usic, Thomas M. S. Wolever, Walter C. Willett, David J. A. Jenkins

**Affiliations:** 1 Epidemiology and Biostatistics Unit, Istituto Nazionale Tumori-IRCCS-“Fondazione G. Pascale”, 80131 Napoli, Italy; 2 Section of Nutrition and Dietetics, Division of Medicine, Department of Clinical Service, Oslo University Hospital, 0424 Oslo, Norway; a.m.aas@medisin.uio.no; 3 Institute of Clinical Medicine, University of Oslo, 0318 Oslo, Norway; 4 Department of Nutrition, Exercise and Sports (NEXS) Faculty of Science, University of Copenhagen, 2200 Copenhagen, Denmark; ast@nexs.ku.dk; 5 School of Life and Environmental Sciences, The University of Sydney, 2006 Sydney, Australia; fiona.atkinson@sydney.edu.au (F.S.A.); jennie.brandmiller@sydney.edu.au (J.C.B.-M.); 6 Charles Perkins Centre, The University of Sydney, 2006 Sydney, Australia; 7 Oldways, Boston, MA 02116, USA; sara@oldwayspt.org; 8 Accredited Practising Dietitian, 2006 Sydney, Australia; alan@dralanbarclay.com; 9 Department of Food and Drug, University of Parma, 43120 Parma, Italy; furio.brighenti@unipr.it; 10 Departament de Bioquímica i Biotecnologia, Unitat de Nutrició, Universitat Rovira i Virgili, 43201 Reus, Spain; monica.bullo@urv.cat (M.B.); jordi.salas@urv.cat (J.S.-S.); 11 Human Nutrition Unit, University Hospital of Sant Joan de Reus, Institut d’Investigació Sanitària Pere Virgili (IISPV), 43201 Reus, Spain; 12 Centro de Investigación Biomédica en Red Fisiopatología de la Obesidad y la Nutrición (CIBEROBN), Institute of Health Carlos III, 28029 Madrid, Spain; 13 Institute of Nutrition, Consumption and Health, Faculty of Natural Sciences, Paderborn University, 33098 Paderborn, Germany; anette.buyken@uni-paderborn.de; 14 IRCCS MultiMedica, Diabetes Department, Sesto San Giovanni, 20099 Milan, Italy; antonio.ceriello@hotmail.it; 15 Biopolymers Group, Departments of Biochemistry and Nutritional Sciences, Faculty of Life Sciences & Medicine, King’s College London, Franklin-Wilkins Building, 150 Stamford Street, London SE1 9NH, UK; peter.r.ellis@kcl.ac.uk; 16 Spinney Nutrition, Shirwell, Barnstaple, Devon EX31 4JR, UK; nutrition@thespinney.co.uk; 17 Clinical Nutrition Research Centre, Singapore Institute for Clinical Sciences, Singapore 637551, Singapore; jeya_henry@sics.a-star.edu.sg; 18 Departments of Nutritional Science and Medicine, Faculty of Medicine, University of Toronto, Toronto, ON M5S 1A8, Canada; cyril.kendall@utoronto.ca (C.W.C.K.); john.sievenpiper@utoronto.ca (J.L.S.); thomas.wolever@utoronto.ca (T.M.S.W.); david.jenkins@utoronto.ca (D.J.A.J.); 19 Clinical Nutrition and Risk Factor Modification Centre, St. Michael’s Hospital, Toronto, ON M5C 2T2, Canada; 20 College of Pharmacy and Nutrition, University of Saskatchewan, Saskatoon, SK S7N 5B5, Canada; 21 Department of Clinical Sciences and Community Health, Università degli Studi di Milano, 201330 Milan, Italy; carlo.lavecchia@unimi.it; 22 Department of Epidemiology and Medicine, Brown University, Providence, RI 02912, USA; simin_liu@brown.edu; 23 Independent Nutrition Logic Ltd., 21 Bellrope Lane, Wymondham NR180QX, UK; glivesey@inlogic.co.uk; 24 Nutrition Foundation of Italy, Viale Tunisia 38, I-20124 Milan, Italy; poli@nutrition-foundation.it; 25 Department of Clinical Medicine and Surgery, Federico II University, 80147 Naples, Italy; riccardi@unina.it; 26 Department of Public Health and Caring Sciences, Clinical Nutrition and Metabolism, Uppsala University, 751 22 Uppsala, Sweden; ulf.riserus@pubcare.uu.se; 27 Institute of Cardiometabolism and Nutrition, ICAN, Pitié Salpêtrière Hospital, F75013 Paris, France; salwa.rizkalla@psl.aphp.fr; 28 Division of Endocrinology and Metabolism, Department of Medicine, St. Michael’s Hospital, Toronto, ON M5C 2T2, Canada; 29 Li Ka Shing Knowledge Institute, St. Michael’s Hospital, Toronto, ON M5C 2T2, Canada; 30 Hellenic Health Foundation, Alexandroupoleos 23, 11527 Athens, Greece; atrichopoulou@hhf-greece.gr; 31 Glycemic Index Foundation, 2037 Sydney, Australia; kathyu@gifoundation.org.au; 32 Departments of Nutrition and Epidemiology, Harvard T. H. Chan School of Public Health and Harvard Medical School, Boston, MA 02115, USA; wwillett@hsph.harvard.edu

**Keywords:** dietary fibre, labelling, carbohydrate quality, ICQC, consensus

## Abstract

Dietary fibre is a generic term describing non-absorbed plant carbohydrates and small amounts of associated non-carbohydrate components. The main contributors of fibre to the diet are the cell walls of plant tissues, which are supramolecular polymer networks containing variable proportions of cellulose, hemicelluloses, pectic substances, and non-carbohydrate components, such as lignin. Other contributors of fibre are the intracellular storage oligosaccharides, such as fructans. A distinction needs to be made between intrinsic sources of dietary fibre and purified forms of fibre, given that the three-dimensional matrix of the plant cell wall confers benefits beyond fibre isolates. Movement through the digestive tract modifies the cell wall structure and may affect the interactions with the colonic microbes (e.g., small intestinally non-absorbed carbohydrates are broken down by bacteria to short-chain fatty acids, absorbed by colonocytes). These aspects, combined with the fibre associated components (e.g., micronutrients, polyphenols, phytosterols, and phytoestrogens), may contribute to the health outcomes seen with the consumption of dietary fibre. Therefore, where possible, processing should minimise the degradation of the plant cell wall structures to preserve some of its benefits. Food labelling should include dietary fibre values and distinguish between intrinsic and added fibre. Labelling may also help achieve the recommended intake of 14 g/1000 kcal/day.

## 1. Introduction

**Figure 1:**
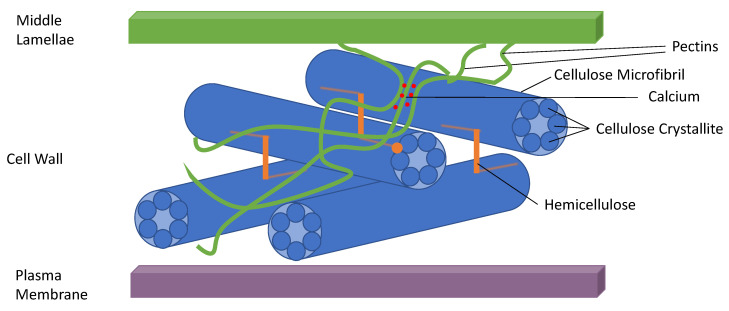
Carbohydrate components of a primary plant cell wall. A cartoon of the carbohydrate components of a primary plant cell wall demonstrating the supramolecular nature of the wall and the diversity of the cell wall constituents which contribute to dietary fibre. The cellulose microfibrils are composed of crystallites which are further composed of cellulose chains. The cellulose microfibrils are stacked upon one another to give strength as the skeleton of the wall. Hemicellulose is thought to keep the microfibrils apart. The nature of hemicellulose present varies considerably between plants. Pectin is a mega molecule, used for water transport throughout the plant. There are various different sections within pectin, the proportions vary between plants. The egg box region is shown here where different strands of pectin are bound together by calcium. There is a high concentration of pectins in the middle lamellae which interact with the neighbouring cell walls [4].

Conceptually, dietary fibre is a generic term describing non-absorbed plant carbohydrates and relatively small amounts of associated non-carbohydrate components (e.g., phenolic compounds, waxes, and proteins) that are not digested by endogenous enzymes or absorbed in the human small intestine [1,2]. Some forms of dietary fibre are digested by intestinal bacterial enzymes and utilised as substrates for growth and metabolism. The main contributors of fibre to the diet are the cell walls of plant tissues, which are supramolecular polymer networks containing variable proportions of cellulose, hemicelluloses, pectic substances, and the non-carbohydrate components, such as the phenolic compound lignin (Figure 1). Other sources of fibre in the diet include fructans (e.g., inulins), which are not part of the plant cell walls but are synthesised and stored in the cell vacuole [3,4].

**Figure 2:**
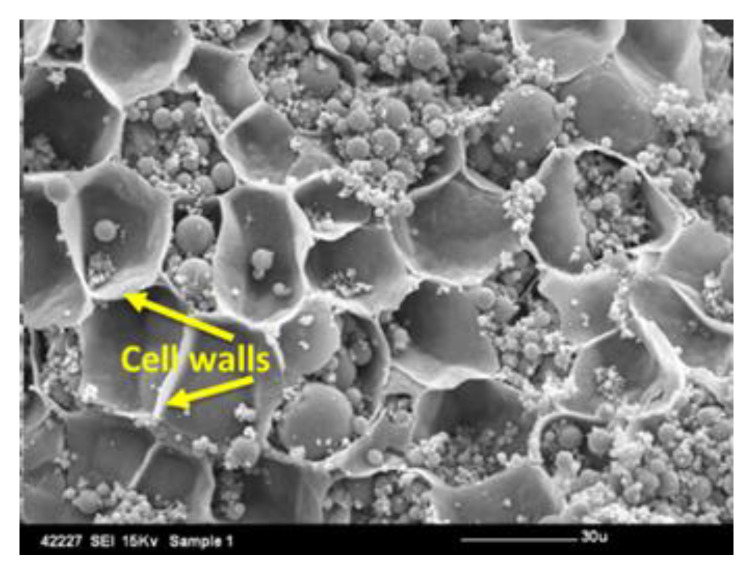
Surface of an almond seed post-mastication showing ruptured cell walls (dietary fibre). Micrograph, produced by scanning electron microscopy, of the surface of a masticated particle of almond seed. The cell walls (dietary fibre) have been ruptured (as marked by arrows) by chewing, exposing the nutrients inside the cells of the almond cotyledon tissue. Many of these cells still contain protein and lipid (oil bodies and coalesced oil droplets), which are potentially available for digestion (i.e., bioaccessible). Nutrients in intact cells below the fractured surface are not bioaccessible because the dietary fibre acts as a physical barrier to digestion. The scale bar is 30 μm.

However, there is much that is not known about dietary fibre, in part because the structure of the plant cell wall, which makes up the majority of our dietary fibre, has not been fully elucidated. In turn, the overall structure of the polymers, and how they interact with each other within the plant cell wall, is not yet fully understood [3,4]. Added to this, what occurs to the matrix of the cell wall during chewing (Figure 2) and movement through the digestive tract is not clear [5], and a substantial percentage of dietary fibre is digested by the microbes in the colon. The nature and actions of the microbiome are just beginning to be explored [6].

## 2. Definitions

There is still disagreement about the definition of dietary fibre and how this very complex array of plant materials should be analysed. Current definitions are typically based around descriptions provided by national and international bodies for food standards, such as CODEX Alimentarius, and have focused on fibre being the non-digested and/or non-absorbed fraction of food carbohydrates derived from plants [7,8,9,10,11]. Dietary fibre definitions around the world have been summarised (10), and countries adopting the CODEX definition include Australia, Canada, China, the European Union, Malaysia, New Zealand, and the USA, among others. The US Food and Drug Administration issued a position paper in 2018 on what constitutes dietary fibre for food labelling purposes [11].

It may be useful to distinguish between dietary fibre, as plant cell walls (the main source of fibre) that are part of the plant food matrix, and fibre supplements that are added to food products for a specific physiological/health outcome (e.g., laxation, cholesterol-lowering, and prebiotic activity) [5]. The term natural fibre may be better described as dietary fibre that is intrinsically part of the cell wall material in edible plants such as fruits, vegetables, cereals, nuts, pulses, and even seaweed in some diets (from now on defined as intrinsic fibre). The intrinsic fibre may be modified when processed commercially and/or domestically and may not have the same physiological and metabolic effects of the original intrinsic fibre. These include the purified fibres derived from cereals (e.g., mixed-linkage β-glucans from barley and oats, among others). Some commercially available types of fibre are semi-synthetic, such as hydroxypropyl methylcellulose, which is a chemically modified cellulose. These may also be called novel types of dietary fibre in certain countries (e.g., Canada).

Another distinction seen in the literature is insoluble versus soluble fractions of fibre, which are classified by chemical analysis but not based on their functional behaviour in vivo [5]. These fractions are based on early attempts to classify fibre according to their dissolution properties in aqueous media in the laboratory. There are different chemical methods used for determining dietary fibre (e.g., the gravimetric AOAC method and GC analysis of non-starch polysaccharides) and values do vary significantly, as do the values for ‘soluble’ and ‘insoluble’ fractions. These broad classifications continue to be used in nutrition and public health literature, despite their limited use in providing information about functional properties in the gut, and their specific effects on metabolism. Solubility and viscosity are terms often used interchangeably to describe the same type of fibre; however, a soluble fibre that dissolves in aqueous media may not be viscous. Water-soluble types of fibre have the ability to lower fasting blood cholesterol and postprandial glycaemia [12]. These metabolic effects are linked to the capacity of soluble fibre to increase digesta viscosity and slow down the digestion of starch and other macronutrients. The viscosity-enhancing property of a soluble fibre is highly dependent on its polymer concentration and molecular weight, assuming it has solubilised in the gut.

## 3. Health Benefits

Dietary fibre can modify gastrointestinal function from the mouth to the anus. The specific physiological effects depend, crucially, on the physico-chemical properties of individual plant polysaccharides and oligosaccharides, and also on the structural integrity of fibre as cell walls, which is an important part of the architecture of the plant tissue [5]. These effects may include increasing or decreasing salivation, luminal viscosity, the gastric emptying rate, nutrient digestion and absorption, transit time, faecal bulking, laxation, fermentation, colonic pH, microbiota amount and composition, and binding of mucus, enzymes, bile acids and other metabolites, which may also be bioactive [13].

Beyond the gut, the established metabolic effects include the lowering of blood cholesterol and postprandial blood glucose, and fasting blood glucose in patients with diabetes [12]. In particular, these effects have been observed with isolated viscous fibres, such as psyllium, mixed-linkage β-glucans, guar gum (galactomannan), glucomannan, and pectic polysaccharides [14]. Another plant isolate, inulin-type fructans, though non-viscous, can lower fasting glucose and insulin and fasting LDL-cholesterol while increasing HDL-cholesterol in patients with diabetes, and to a lesser extent in overweight and obese persons [15]. A manufactured low-viscosity, digestion-resistant maltodextrin also lowers postprandial and fasting blood glucose from drinks and solid foods [16]. The molecular weight of the extracted viscous polysaccharide influences the effectiveness of the metabolic responses [9]. These observations implicate fibre as capable of modifying metabolism. Moreover, fibre-rich sources of edible plants—such as pulses, nuts, barley, oats, and some vegetables and fruits—have been shown to improve long-term control of established cardio-metabolic risk factors, i.e., blood lipids, blood glucose, blood pressure, and body weight. Many of these beneficial health effects have been attributed to the presence of fibre in these foods.

Prospective cohort studies have reported inverse associations between total dietary fibre intake and body weight, risk of type 2 diabetes, cardiovascular disease, stroke, some types of cancer, and total mortality. These associations have been shown with fibre intake from grains, legumes, nuts, fruit, and vegetables. The associations are independent of the dietary glycaemic index and glycaemic load, the effects of which are additive, at least for reducing the risk of diabetes from both observational and interventional studies [17,18]. However, despite the intensive research on nutritional epidemiology, many questions on the role of fibre in disease remain unanswered, and the contribution of associated substances to causality has been difficult to prove. Thus, the associations with fibre seen in epidemiological studies may be partially due to associated components, such as some amino acids, unsaturated fat, minerals, vitamins, and some phytochemicals, such as polyphenols, phytosterols, and phytoestrogens. In nutrition, a distinction needs to be made between intrinsic sources of dietary fibre and purified or chemically/physically modified forms of fibre, given that the three-dimensional (3D) matrix of the plant cell wall confers benefits above fibre isolates. This is because cell walls, and the 3D matrix of the plant cell walls, affect the functional properties of ‘fibre’ impacting on the digestibility of the cell contents [5]. This may be part of the reason for the strong benefits seen in wheat fibre in cohort studies, despite the lack of effect seen in the short-term clinical trials for cardiovascular risk factors [19,20,21,22]. In randomised controlled trials comparing refined and wholegrain cereal foods, when the particle size of the fibre is made too small, the plant cell wall integrity and tissue architecture may be lost. When tissue and the cell wall 3D matrix are sufficiently intact, it can lead to nutrients being slowly absorbed or even not absorbed. For example, cereal foods with a substantially intact tissue structure can also contribute starch as a source of a slowly and/or non-digestible food carbohydrate [23,24,25].

Fibre in wholefoods, isolates, and modified forms can be sources of substrate for micro-organisms in the large intestine, affecting the amount and species composition of the microbiota and their collective functional capacity to improve the health of the gut and other organs via modulation of the immune system, production of bioactive metabolites (e.g., short-chain fatty acids), and the reduction of intracolonic pH, with beneficial effects on the colonic mucosa and blood lipid levels [26].

At the population level, we suggest replacing some animal foods, and high glycaemic index foods containing refined starches and sugars, with slowly digestible carbohydrate foods with a low glycaemic index that are rich in fibre. This would have a favourable impact on glycaemic control and, hence, diabetes, cardio-metabolic risk, and possibly some diabetes-related cancers [27]. Minimising the degradation of the plant cell wall structures and tissue architecture is important where slow digestibility of macronutrients, such as starch, is required for the production of healthy foods, and also in the development of low glycaemic index foods. These issues are important, especially in some parts of the world with a high risk of cardio-metabolic disease, where dietary fibre intake tends to be below the recommended intake levels. However, it is recognised that in foods where mineral bioavailability needs to be increased, the rupture of the cell walls may provide a way to improve mineral status, e.g., a higher iron bioavailability through the micro-milling of wheat aleurone [28].

Much research is still required to fully understand the physiological and nutritional effects of dietary fibre. We need to further understand the interaction of fibre with the microbiota, and we also need to understand more about the structure, physico-chemical properties, and composition of dietary fibre. Additionally, we require an improved mechanistic insight into how the components associated with dietary fibre interact with fibre, and the impact on metabolic outcomes. Furthermore, an improved understanding is required on the role played by the 3D architecture of dietary fibre on nutrient release (i.e., bioaccessibility), fermentability by gut bacteria, prebiotic activity, and the roles these have in human health. When these are better elucidated, there will be a need to communicate to food producers, consumers, and health professionals on how to make better food choices [5].

Certain types of dietary fibre affect the amount and composition of microbiota, which has been studied mostly in regard to fermentative micro-organisms in the large intestine. Inulins, found in plants like chicory root and galacto-oligosaccharides, present in or from milk, are prime examples of non-digestible carbohydrate or dietary fibres that, among others, behave as prebiotics [29,30,31]. A prebiotic was recently defined by consensus as “a substrate that is selectively utilised by host micro-organisms conferring a health benefit” [32]. Putative health benefits include the inhibition of pathogens reaching the large intestine, immune stimulation, improved cardiometabolic status, improved mental health, and support to bone mineralisation, among others [32]. More long-term randomized controlled trials are needed to establish causality, which appears promising, though prebiotic effects may not be seen in everyone, especially in persons already in good health or having a sufficient amount and composition of beneficial micro-organisms. Moreover, not all dietary fibres are prebiotic, but the effect prebiotic fibre has can depend on the amount of other dietary fibre that is consumed [33].

Many chemical/enzyme methods exist for analysing dietary fibre, but those used for labelling are often different from those used in food composition tables. Current analytical methods reflect a heterogeneous mix of chemical entities, with no information on any subspecies of fibre or any information on the structural characteristics of the fibre present. One example of how dietary fibre is measured is by using the AOAC enzyme-gravimetric method, which is intended to simulate the physiological conditions of digestion, and measures all the components of fibre, as currently defined by CODEX Alimentarius. This kind of analysis is limited when being used to interpret mechanistic data on the functional properties of cell walls, individual cell wall polysaccharides and storage oligosaccharides. More informative methods, notably dissolution kinetics, molecular weight of individual polysaccharides, and cell wall porosity are urgently required for characterising dietary fibre in nutritional and epidemiological studies, if food sources of dietary fibre for health are to be optimised.

## 4. Recommendations to the Public and to Health Professionals

It is generally agreed that dietary fibre is an important part of a sustainable, balanced, healthy diet [34]. Consumption of dietary fibre is below the recommended intake levels for optimal health in many parts of the world and may be decreasing. We recommend maintaining or increasing dietary fibre intake to the recommended levels.

We support the Institute of Medicine recommendations for the total dietary fibre of 14 g/1000 kcal/day. We suggest that this should mainly come from intrinsic dietary fibre. Data from cohort studies with intakes beyond this amount are limited, but many traditional societies consume larger amounts and have a lower risk of chronic diseases.

## 5. Recommendations to the Food Industry

The food industry plays an important role in developing new food ingredients and products that have public health benefits and are also highly palatable. In developing new high-fibre foods, the sensory characteristics are important and will strongly influence whether people consume them. At the same time, if these do not have nutritional benefits then such products would be of little nutritional value, regardless of how technologically innovative they may be. It is important to recognise that increasing the fibre content on the food label does not guarantee any enhanced nutritional benefits in a product.

Recommendations to the food industry would depend on the reasons why particular types of fibre are being added, and how they are processed, given that mechanical and hydrothermal processing can affect their properties. For example, in wheat grain there is an advantage in preserving some of the structural integrity of the cell walls of the starch-rich endosperm, in order to produce flour that is digested more slowly and has a beneficial impact on postprandial glycaemia (23). However, if the health outcome is to improve the iron bioavailability in wheat, then there may be advantages to micro-milling (rupturing) the aleurone cell layer, which has a high iron concentration (28). In producing foods for the general population, the first example would be the most appropriate recommendation while, for populations with nutritional deficiencies, the second recommendation may preferentially apply. Therefore, we generally encourage the food industry to preserve many of the benefits of dietary fibre rich foods by minimising the degradation of the plant cell wall structures and tissue architecture, while maintaining palatability, except in situations of special dietary requirements and specific physiological or clinical outcomes (e.g., the use of prebiotic oligosaccharides and viscous polysaccharides). 

Currently, labelling the dietary fibre content of foods in certain countries around the world, including Europe, is not mandatory. This represents a problem for consumers, researchers, and medical staff dealing with patient diets. We support the mandatory use of fibre on food labels.

Labelling should distinguish between fibre that is endogenous to foods and that added as a functional supplement because synthetic or purified fibre will not be accompanied by the micronutrients and phytochemicals in foods and, thus, may not predict the same health outcomes. Functional (or other) supplemental fibre, where permitted, should be listed separately among ingredients. The labelling of dietary fibres could be of the form “FIBRE N g PER 100 g, of which X g is SUPPLEMENTAL”.

## 6. Conclusions

Dietary fibre and its associated non-carbohydrate components have been inversely associated with disease outcomes. Food labelling should include dietary fibre, and distinguish between intrinsic and purified added fibre, given that the intact plant cell wall may confer benefits beyond fibre isolates. The labelling of dietary fibre may also help to achieve the recommended intake of 14 g/1000 kcal/day for health benefits. To extend these recommendations, further studies on the interrelation of dietary fibre, prebiotics, and health, which aim to optimise both the health potential of foods and related food processing methods, are advised. This would include how the structures and the 3D matrix, composition, and physico-chemical properties of dietary fibre affect digestion, gastrointestinal function, and the role of the microbiome.
